# Evaluation of Full-Length Versus V4-Region 16S rRNA Sequencing for Phylogenetic Analysis of Mouse Intestinal Microbiota After a Dietary Intervention

**DOI:** 10.1007/s00284-022-02956-9

**Published:** 2022-07-30

**Authors:** Saeed Katiraei, Yahya Anvar, Lisa Hoving, Jimmy F. P. Berbée, Vanessa van Harmelen, Ko Willems van Dijk

**Affiliations:** 1 Department of Human Genetics, Leiden University Medical Center, Leiden, The Netherlands; 2 Einthoven Laboratory for Experimental Vascular Medicine, Leiden University Medical Center, Leiden, The Netherlands; 3 Division of Endocrinology, Department of Medicine, Leiden University Medical Center, Leiden, The Netherlands; 4 Department of Human Genetics, Leiden Genome Technology Center (LGTC), Leiden University Medical Center (LUMC), Leiden, The Netherlands

## Abstract

The composition of microbial communities is commonly determined by sequence analyses of one of the variable (V) regions in the bacterial 16S rRNA gene. We aimed to assess whether sequencing the full-length versus the V4 region of the 16S rRNA gene affected the results and interpretation of an experiment. To test this, mice were fed a diet without and with the prebiotic inulin and from cecum samples, two primary data sets were generated: (1) a 16S rRNA full-length data set generated by the PacBio platform; (2) a 16S rRNA V4 region data set generated by the Illumina MiSeq platform. A third derived data set was generated by in silico extracting the 16S rRNA V4 region data from the 16S rRNA full-length PacBio data set. Analyses of the primary and derived 16S rRNA V4 region data indicated similar bacterial abundances, and α- and β-diversity. However, comparison of the 16S rRNA full-length data with the primary and derived 16S rRNA V4 region data revealed differences in relative bacterial abundances, and α- and β-diversity. We conclude that the sequence length of 16S rRNA gene and not the sequence analysis platform affected the results and may lead to different interpretations of the effect of an intervention that affects the microbiota.

**Supplementary Information:**

The online version contains supplementary material available at 10.1007/s00284-022-02956-9.

## Introduction

The composition of gut microbiota have been associated with a variety of pathophysiological conditions, including obesity, low-grade inflammation, and overt disease [1–3]. We [4–6] and others [7, 8] have exploited possibilities to beneficially affect microbiota using probiotics or dietary compounds that affect the composition and/or activity of the gut bacteria. To determine the success of intervention, the composition of gut microbiota are commonly determined by massive parallel sequencing of one of the variable (V) regions of the bacterial 16S rRNA gene [9]. Sequence analysis of the V-region of 16S rRNA has proven to be a powerful tool to describe the composition of bacterial communities [10, 11]. However, the resolution of the taxonomic description of the communities is limited by the uniqueness of the V-region sequences and available reference databases [12]. Numerous different bacterial species have almost identical V-region sequences which makes distinguishing of these bacteria based on a single V-region impossible. The currently available 16S reference databases that are used for taxonomic classification of 16S sequencing data are still quite limited and do not contain a reference sequence for all experimentally obtained 16S sequences [13, 14]. Therefore, some 16S V-region sequences can only be assigned up to the family and/or genus level or cannot be assigned at all.

Massive parallel sequencing of 16S rRNA V-regions has been made possible by the development of next-generation sequencing technology (NGS). A typical NGS run on an Illumina MiSeq will provide several million 250 bp paired-end reads per flow cell. The advantage of high throughput is countered by the relatively short reads that are produced by NGS. Although many of the limitations of short-read sequencing can be addressed using computational approaches, it is extremely challenging, if not impossible, to assemble longer sequences composed of highly homologous parts. Examples of this are repeated sequences in the human genome, but also the repeated sequences in the genomes of various bacteria that constitute the microbiota. A number of so-called third-generation sequencing technologies have been developed to overcome these limitations by sequencing very long amplicons. One such approach is developed by Pacific BioSciences (PacBio) and is termed single-molecule real-time (SMRT) sequencing [15].

We aimed to assess whether sequencing the full-length 16S rRNA gene using SMRT sequencing affected the results and interpretation of a dietary intervention compared to sequencing only the V4 region of this gene. This study included two experimental conditions; a Western-type diet (WTD) and a WTD complemented with the fibre inulin. Inulin is a fructose polymer that can only be degraded by intestinal microbiota and therefore strongly favours the expansion of specific intestinal microbiota [16–19]. To compare the effects of the dietary intervention measured on either the PacBio or Illumina MiSeq platform, we performed taxonomic analysis and diversity analysis on primary and derived data sets.

## Materials and Methods

### Cecum Samples

Cecum content was collected for microbial analysis. The cecum samples used in study were obtained in the context of a larger study of which the results were published recently [5].

### DNA Isolation

From cecum samples, genomic DNA was extracted using phenol: chloroform: isoamyl alcohol (25: 24: 1) (Invitrogen), precipitated with isopropanol, and washed with 70% ethanol.

### PacBio Sequencing

16S rRNA full-length amplification was performed using degenerate primers containing 5’ M13 universal tail sequences (Table S1). The 16S locus was amplified using LA Taq polymerase (Takara) with 400 μM dNTPs, 50 ng DNA template, and 400 nM of each primer in 1 × LA buffer + magnesium with 30 cycles of PCR (20 s 94 °C, 30 s 48 °C, 2 min 68 °C). PCR reactions were size selected using 0.65 × AMPure XP beads (Beckman Coulter). Amplicons were barcoded in a second PCR reaction containing universal tail oligos complementary to the M13 universal tail sequences (Table S1). Barcodes were added using Herculase II Taq polymerase (Agilent) with 250 μM dNTPs, 2ul of purified PCR product, and 400 nM of each primer in a 1 × reaction buffer with 5 cycles of PCR (20 s 95 °C, 20 s 58 °C, 2 min 72 °C). The barcoded amplicons were size selected using 0.65 × AMPure XP beads (Beckman Coulter). 500 ng of barcoded amplicons were prepared for sequencing using the amplicon template preparation protocol, 2015 release (Pacific Biosciences) including DNA damage repair and SMRTbell adapter ligation. Libraries were sequenced on the Pacific Biosciences RSII using MagBead loading with 6 h of movie time and P6-C4 chemistry.

### In Silico Isolation of V4 Regions from Full-Length 16S rRNA PacBio Sequencing Data

V4 regions from full-length 16S rRNA PacBio data set were in silico isolated by the V-ripper script [20] using forward primer (5′‐GTGCCAGCMGCCGCGGTAA‐3′) and the reverse primer (5′‐GGACTACHVGGGTWTCTAAT‐3′). Subsequently, isolated sequences with length between 100 and 300 bp were retained.

### Illumina Sequencing

Genomic DNA was sent to the Broad Institute of MIT and Harvard (Cambridge, USA). Microbial 16S rRNA was amplified targeting the hyper‐variable V4 region using forward primer 515F (5′‐GTGCCAGCMGCCGCGGTAA‐3′) and the reverse primer 806R (5′‐GGACTACHVGGGTWTCTAAT‐3′). The cycling conditions consisted of an initial denaturation of 94 °C for 3 min, followed by 25 cycles of denaturation at 94 °C for 45 s, annealing at 50 °C for 60 s, extension at 72 °C for 5 min, and a final extension at 72 °C for 10 min. Sequencing was performed using the Illumina MiSeq platform generating paired‐end reads of 175 bp in length in each direction. Overlapping paired‐end reads were subsequently aligned. Details of this protocol have previously been described [21].

### Sequencing Data Analysis

All three data sets were analysed using the operational taxonomic unit (OTU) approach. This was done by using the QIIME pipeline [22]. We used SILVA 132 QIIME release as reference OTU taxonomy database. Prior to OTU picking, each data set was quality filtered by sickle version 1.33 and low-quality reads were discarded. Open reference OTU picking strategy with 97% sequence similarity and minimum OTU size of two reads was used. The α-diversity metric based on observed OTUs was calculated continuously from 50 reads/sample up to 3300 reads/sample with increasing steps of 50 reads, with 10 × rarefaction. Unweighted UniFrac distances, with 10 jack-knifed replicates was measured at rarefaction depth of 3000 reads per sample, based on the unfiltered OTU table and relative bacterial abundance was determined. Prior to relative abundance visualization, rare taxa that were present at less than 0.1% were filtered. Sequence data are submitted to SRA database and are accessible with BioProject accession number PRJNA786882.

## Results

### Sequencing Depth

Cecum content from mice fed a WTD without or with 10% inulin for 11 weeks was collected (*n* = 2 per group) and genomic DNA was extracted. The full-length 16S rRNA gene was amplified for PacBio sequencing, and the V4 region of the bacterial 16S rRNA gene was PCR amplified for Illumina short-read sequencing. To determine platform bias in the data sets obtained from the PacBio and Illumina platforms, a 16S rRNA V4 region data set was generated in silico from the full-length 16S rRNA PacBio data set (V4 PacBio). Table S2 shows that the read count obtained by PacBio and Illumina sequencing are in range of a typical run for the platforms, and the reads have the correct mean read length for the full-length 16S rRNA (approx. 1500 bp) and V4 region (approx. 250 bp). Interestingly, the V4 PacBio read count for individual samples are approximately 50% of the read count for the full-length 16S rRNA PacBio data they were derived from (Table S2). The V-ripper script in combination with the used primer sequences, apparently, does not recognize 50% of the full-length 16S rRNA sequences.

### Sequencing Data Analysis

For operational taxonomic unit (OTU) picking, open reference OTU picking strategy with 97% sequence similarity and minimum OTU size of two reads was used. The minimum OTU size of at least two sequences/OTU ensured that singletons are excluded from the data. Table 1 shows the number of OTUs for individual samples and the number of sequences that these OTUs contained. In the 16S rRNA full-length PacBio data set, proportionally more reads were discarded in the OTU picking step compared to both 16S rRNA V4 data sets. These discarded reads were singletons and sequences that failed to align with the reference database. Furthermore, sequencing full-length 16S rRNA resulted in a higher percentage of unassigned taxa (2.9–8.4% of total reads) compared to both V4 data sets (0.05–0.6% of total reads; Table 1). These were reads without any reference sequence available in the reference database. The number of unassigned reads in the full-length 16S rRNA data set was in particular higher for samples of inulin-fed mice compared to samples of control mice.

**Table 1:** Microbial community analysis

Group	Data set	Sample	Read count	OTU count	Reads assigning taxonomic labels (coverage %)				
					Class	Order	Family	Genus	Unassigned
Control	FL	C1	15,261	1821	14,781 (96.9%)	14,781 (96.9%)	14,769 (96.8)	13,455 (88.2%)	480 (3.1%)
	FL	C2	7022	1198	6819 (97.1%)	6819 (97.1%)	6810 (97.0%)	5919 (84.3%)	202 (2.9%)
	V4 PacBio	C1	9195	435	9181 (99.8%)	9181 (99.8%)	9178 (99.8%)	8071 (87.8%)	14 (0.2%)
	V4 PacBio	C2	3979	366	3955 (99.4%)	3955 (99.4%)	3951 (99.3%)	3386 (85.1%)	23 (0.6%)
	V4 Illumina	C1	105,426	1424	105,323 (99.9%)	105,323 (99.9%)	105,174 (99.8%)	87,984 (83.5%)	103 (0.1%)
	V4 Illumina	C2	139,693	1572	139,583 (99.9%)	139,583 (99.9%)	138,285 (99.0%)	114,513 (82.0%)	110 (0.1%)
Inulin	FL	In1	13,481	2199	12,986 (96.3%)	12,986 (96.3%)	12,968 (96.2%)	10,098 (74.9%)	492 (3.6%)
	FL	In2	5350	1056	4900 (91.6%)	4900 (91.6%)	4780 (89.3%)	3131 (58.5%)	449 (8.4%)
	V4 PacBio	In1	9609	618	9595 (99.9%)	9595 (99.9%)	9568 (99.6%)	6919 (72.0%)	14 (0.1%)
	V4 PacBio	In2	3309	353	3290 (99.4%)	3290 (99.4%)	3181 (96.1%)	2060 (62.3%)	19 (0.6%)
	V4 Illumina	In1	144,897	2036	144,761 (99.9%)	144,761 (99.9%)	144,228 (99.5%)	85,122 (58.7%)	135 (0.1%)
	V4 Illumina	In2	198,271	2036	198,171 (99.9%)	198,171 (99.9%)	195,756 (98.7%)	96,617 (48.7%)	99 (0.05%)

### Full-Length 16S rRNA Results into Higher α-Diversity

**Fig. 1:**
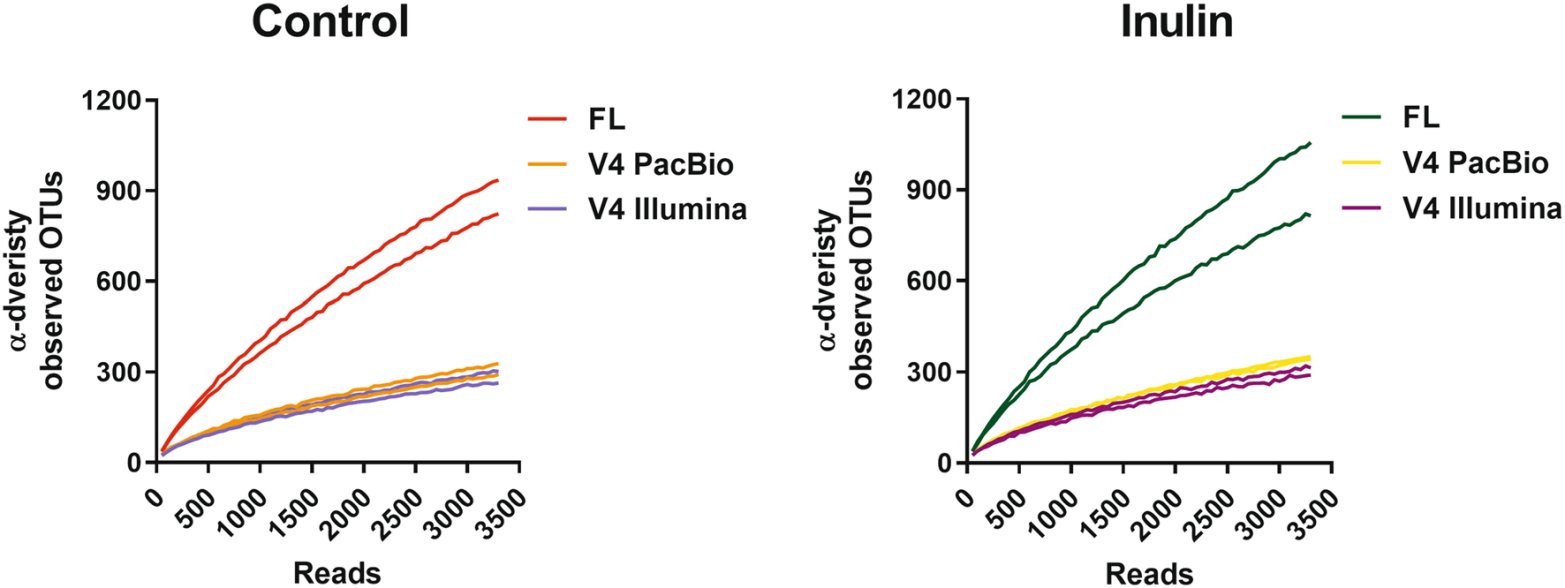
α-diversity. α-diversity metric observed species was calculated continually both for control and inulin-fed mice (*n* = 2) with 10 × rarefaction from 50 reads/sample up to 3300 reads/sample with steps of 50 reads. Each line represents one individual sample. FL, 16S rRNA full-length PacBio

The OTU richness was assessed by plotting α-diversity versus sequencing depth. The α-diversity expressed as number of unique observed OTUs was calculated continuously from 50 reads/sample up to 3300 reads/sample with increasing steps of 50 reads, with 10 × rarefaction. Already at a sequencing depth of 300 reads/sample, α-diversity of 16S rRNA full-length PacBio samples was increased compared to both V4 PacBio and V4 Illumina data sets for control and inulin-fed samples (Fig. 1), while α-diversity of the V4 PacBio and V4 Illumina data sets were comparable. These data show that sequencing the full-length 16S rRNA resulted in a higher number of unique OTUs, already at a relatively low sequencing depth.

### Using Full-Length 16S rRNA Reveals a Different Bacterial Phylogeny as Compared with V4 Region

**Fig. 2:**
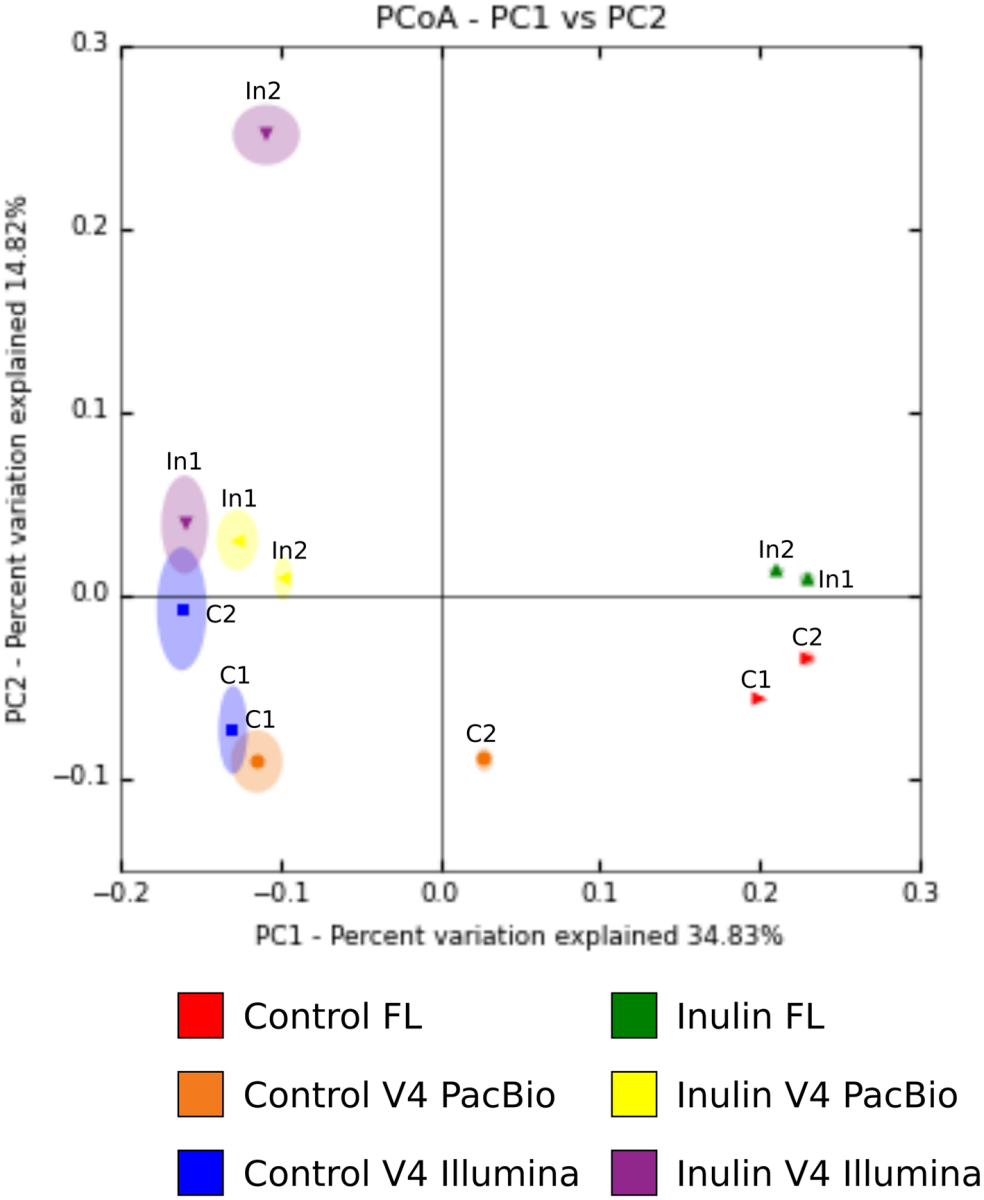
β-diversity, unweighted UniFrac distances. Unweighted UniFrac distances for individual samples were calculated both for control and inulin-fed mice (*n* = 2) using PacBio and Illumina MiSeq platform. Identical sample names in the graphs indicate individual mouse samples studied using different approaches. 10 × Jack-knifing at 3000 reads/sample was performed. C1 and C2 are individual samples from the control group. In1 and In2 are individual samples from the inulin group. FL, 16S rRNA full-length PacBio

The between sample diversity, or β-diversity, was determined by calculating unweighted UniFrac distances. This is a validated and widely used quantitative distance metric for studying microbial community clustering that takes the phylogeny of communities into account [23, 24]. Principal coordinate analysis was performed and the variation explained by the first two principal coordinates is plotted in (Fig. 2). Principal coordinate (PC)1, which explains 34.8% of the data, clearly separates the full-length 16S rRNA PacBio data from the V4 amplicon data. The unweighted UniFrac distance for the V4 PacBio data set was comparable with the UniFrac distance of Illumina V4 regions, indicating limited sequencing platform bias in determining β-diversity. In order to assess the robustness of the UniFrac distance 10 × jack-knifing at 3000 reads/sample was performed for all samples. The jack-knifing variance, indicated by the ellipsoids around the data points, was smaller for the full-length 16S rRNA sequenced samples compared to both V4 data sets (Fig. 2). This indicates that a longer amplicon length provided a more robust UniFrac distance assignment.

### Using Full-Length 16S rRNA Gene Results in a Different Bacterial Composition and Relative Abundance

In addition to diversity analyses, we aimed to study if sequence length affected the taxonomic analysis outcome. We hypothesized that a longer amplicon length increased the resolution of the analysis by detecting additional taxa which would not be observed by sequencing the V4 region only. Therefore, we compared the full-length 16S rRNA PacBio samples with the V4 PacBio and V4 Illumina data sets. In this way, we could exclude platform bias and detect the effects of amplicon length on taxonomic analysis after a dietary intervention.

**Fig. 3:**
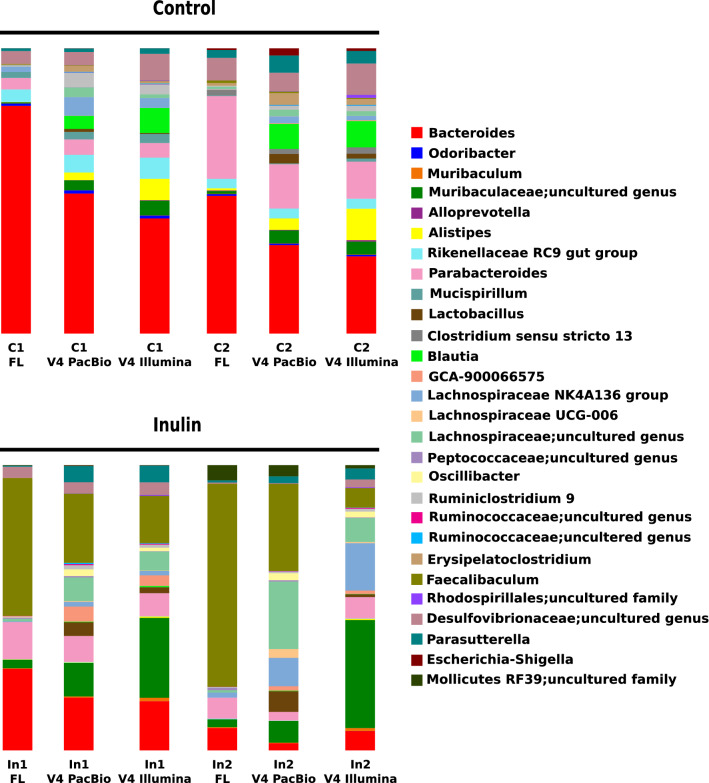
Comparison of microbial composition. Relative bacterial abundance in the cecum content of control and inulin-fed mice (*n* = 2) visualized at genus level. Taxa abundant less than 0.1% of total population are filtered out. C1 and C2 are individual samples from the control group. In1 and In2 are individual samples from the inulin group. FL, 16S rRNA full-length PacBio

Genus level is considered as the maximum resolution of 16S sequencing. Therefore, we compared relative abundance of bacterial taxa in the three data sets at genus level. Sequencing the full-length 16S rRNA gene showed a different relative abundance at genus levels compared to both V4 data sets, both for samples of control and inulin-fed mice (Fig. 3). Bacterial relative abundances of V4 PacBio and V4 Illumina data sets were comparable for control samples. For inulin-fed mice, sample In2 showed variation in relative abundance for several taxa between the V4 PacBio and V4 Illumina data set (Fig. 3). Interestingly, relative abundance of the genus *Faecalibaculum* that blooms with inulin intervention was higher in the full-length 16S rRNA data set compared to both V4 data sets. Relative abundance of the uncultured genus of *Muribaculacea*e family that increases with inulin intervention was lower in the full-length 16S rRNA data set compared to both V4 data sets (Fig. 3). Relative abundance of the *Bacteroides* genus that decreases with inulin intervention was higher in full-length 16S rRNA data set compared to both V4 data sets (Fig. 3). Remarkably, the genus *Lactobacillus* was detected in the V4 PacBio and V4 Illumina data sets for both dietary conditions, but was completely absent inform the full-length 16S rRNA PacBio data set for both dietary conditions. After inulin intervention, other taxa like *GCA-900066575, Lachnospiraceae-UCG006, Lachnospiraceae* uncultured genus, *Oscillibacter* and *Ruminiclostridium 9* were detected in both V4 data sets, and were also almost or completely absent in the full-length 16S rRNA PacBio data set. Taken together, this taxonomic analysis shows that sequencing the full-length 16S rRNA gene results in a different bacterial composition and relative abundance of bacterial species both for control and inulin-fed mice compared to determining the sequence of the V4 region only.

## Discussion

We hypothesized that sequencing the full-length 16S rRNA gene would provide a higher resolution in terms of diversity and taxonomic analyses compared to sequencing a single short amplicon of the 16S rRNA marker gene such as the V4 region.

Our results show that in the in silico-extracted V4 PacBio data set, individual samples have approximately 50% of the read count of the full-length 16S rRNA PacBio data set. This reduction in read count after in silico isolation of the V4 sequences from the full-length 16S rRNA data set might be caused by variability in the primer sequences. It is known that primer choice for sequencing hypervariable regions of 16S rRNA influences sequencing outcome, due to the fact that primers do not cover the 16S rRNA V4 flanking region for all bacteria [25–27]. These data could indicate that a proportion of the taxa that are identified by full-length 16 s rRNA gene sequencing are not detected by sequencing the V4 region only. Alternatively, although the circular consensus sequencing approach of PacBio has a very low error rate, this could also explain a proportion of the V-regions that could not be extracted using the V-ripper script. However, since PacBio sequencing errors are random, this would have no consequences on the distribution and phylogenetic assignment of the extracted sequences.

In addition to primer choice, other factors including the DNA extraction method and choice of the 16S V-region may affect experimental outcome and introduce biases to the diversity and taxonomic analysis. DNA extraction method: Mackenzie et al. studied the effects of different DNA extraction methods, including commercially available DNA isolation kits and the phenol: chloroform: isoamyl alcohol method [28]. Different DNA isolation methods resulted in different DNA yield, DNA quality, and relative abundance of taxon-assigned OTUs. Other studies addressing microbial DNA extraction methods report similar issues [29, 30]. These results emphasize that it is important, if at all possible, to be consistent in the use of a DNA extraction method. Choice of 16S V-region: Sequencing the V4 region in combination with Illumina MiSeq platform has been widely used for taxonomic and diversity analysis [11, 31]. More recently, a combination of two regions like the V2–V3 or V3–V4 region have been used for this purpose [32]. Burkin et al. compared V2–V3 with V3–V4 regions in water samples and reported that V2–V3 sequencing has higher resolution for lower-rank taxa [32]. Abellan-Schneyder et al. conducted an extensive study including six different combinations of the V-regions on human gut and mock samples [33]. They recommended sequencing of V3–V4 regions for human gut samples, but also mentioned that primer choice has significant influence on the resulting microbial composition [33]. Since there seems no consensus on which V-regions provides the best results, investigators should consider the choice for their desired V-region carefully based on the experimental design and sample type. The cecum samples used in study were obtained in the context of a larger study of which the results were published as mentioned in the Materials and Methods section [5]. In order to maintain comparability with previously obtained data we have used the V4 region in this current study.

Diversity analyses and taxonomic analysis are based on OTUs. An OTU is described as a cluster of sequences with a minimum amount of sequence identity; in the case of genus level the threshold for sequence identity is set at 97% similarity [9]. Since OTU picking is based on sequence identity, sequence length can thus affect the number and composition of OTUs in a given data set. α-diversity metric observed that OTUs showed increased number of unique OTUs for the full-length 16A rRNA PacBio data set compared to both V4 data sets.

In addition, our results showed that β-diversity is affected by the sequence length. β-diversity analysis was performed by calculating the unweighted UniFrac distances. The unweighted UniFrac distance is a qualitative distance metric which takes the phylogeny of the sample into account [24]. The PCoA plot of unweighted UniFrac distance is based on the number of shared and unshared branches of the phylogenetic tree of the samples and is therefore a measure of heterogeneity of the bacterial population [23, 24]. Since 16S rRNA full-length PacBio and V4 Illumina sequenced samples are separated in the PCoA plot, we can conclude that these samples had different phylogenetic trees which reflected different bacterial compositions. As samples of the V4 PacBio data set and the V4 Illumina clustered together, we can conclude that the difference in phylogenetic trees and thus bacterial composition is not due to platform bias (PacBio vs Illumina), but caused by the difference in sequence length. Furthermore jack-knifing variance, which determines how often the cluster results are recovered using random subsets of the data, was smaller for the full-length 16S rRNA PacBio samples compared to both V4 data sets and shows that sequencing full-length 16S rRNA resulted in increased robustness of the data [24]. It has previously been shown that the PacBio platform can be used for studying microbiota communities [34, 35]. Based on our findings and the fact that β-diversity metric UniFrac can distinguish bacterial communities at a depth of 50 reads/sample [23], we suggest that the PacBio platform can be used to study intestinal microbial communities at a lower sequencing depth. This allows multiplexing multiple samples on a single-molecule real-time (SMRT) cell in order to reduce resources and sequencing costs.

In addition to diversity analysis, interpretation of experimental outcome requires insight into the bacterial composition of a sample to understand e.g. which bacterial species are able to convert a dietary compound. Taxonomic analysis of the three data sets showed that sequencing full-length 16S rRNA resulted in a different bacterial composition as relative abundances of taxa were increased or decreased with 16S rRNA full-length PacBio after inulin intervention compared to both V4 data sets. Interestingly, the genus *Lactobacillus* was completely absent in the full-length 16S rRNA PacBio data set, while being detected in both V4 data sets. This difference in taxa detection is of major importance for interpretation of biological data. It should be mentioned that in our previous article, exclusively relied on 16S rRNA V4 region sequencing by Illumina, we reported that the genus *Allobaculum* bloomed after inulin intervention [5]. However, here we report that *Faecalibaculum* bloomed after inulin intervention. *Faecalibaculum* is closely related to *Allobaculum* with 86.9% sequence similarity and was recently isolated from laboratory mice [36]. Microbial data of our initial article were analysed using the Greengenes 13.8 reference database and for the current work we used the SILVA 132 reference database which likely explains this discrepancy in annotation.

Sequencing the full-length 16S rRNA gene resulted in the detection of a higher percentage of unassigned reads compared to sequencing the V4 regions only. Interestingly, in our study the percentage of unassigned reads was higher in samples of inulin-fed mice. This finding might suggest that at least part of the bacterial taxa blooming on inulin are in this unassigned fraction of the data. Since we cannot assign these reads, we cannot fully utilize the advantage of full-length 16S rRNA gene sequencing compared to V4 sequencing.

## Conclusion

Taken together, we conclude that sequencing the full-length 16S rRNA gene provides a different view regarding bacterial relative abundance, in-sample diversity, and in in-between-sample diversity, as compared to V4 sequencing regardless of sequence analysis platform. This clearly has implications for interpretation of biological data after a dietary intervention.

## Supplementary Information

Below is the link to the electronic supplementary material.

Supplementary file1 (DOCX 16 kb)

## Declarations

### Research Involving Animals and their Data or Biological Material

Mouse experiments were performed in compliance with Dutch government guidelines and the Directive 2010/63/EU of the European Parliament and had received approval from the University Ethical Review Board (Leiden University Medical Center, The Netherlands).
