# Supplementary material for: Evaluation of Full-Length Versus V4-Region 16S rRNA Sequencing for Phylogenetic Analysis of Mouse Intestinal Microbiota After a Dietary Intervention
Source: Curr Microbiol. 2022 Jul 30;79(9):276. doi: 10.1007/s00284-022-02956-9 (PMC9338901; doi:10.1007/s00284-022-02956-9)
Supplement: Supplementary file 1 — Supplementary file1 (DOCX 16 kb) [file 284_2022_2956_MOESM1_ESM.docx]

Supplementary material for

Evaluation of full-length versus V4-region 16S rRNA sequencing for phylogenetic analysis of mouse intestinal microbiota after a dietary intervention

Saeed Katiraei^1,2^, Yahya Anvar^1,4^, Lisa Hoving^1,2^, Jimmy F.P. Berbée^2,3^, Vanessa van Harmelen^1,2^, Ko Willems van Dijk^1,2,3^*

**Current Micorbiology**

^1^ Department of Human Genetics, Leiden University Medical Center, Leiden, The Netherlands; [s.katiraei@lumc.nl](mailto:s.katiraei@lumc.nl); [S.Y.Anvar@lumc.nl](mailto:S.Y.Anvar@lumc.nl); [L.R.Hoving@lumc.nl](mailto:L.R.Hoving@lumc.nl); [V.J.A.van_Harmelen@lumc.nl](mailto:V.J.A.van_Harmelen@lumc.nl); [V.J.A.van_Harmelen@lumc.nl](mailto:V.J.A.van_Harmelen@lumc.nl)

^2^ Einthoven Laboratory for Experimental Vascular Medicine, Leiden University Medical Center, Leiden, The Netherlands; [J.F.P.Berbee@lumc.nl](mailto:J.F.P.Berbee@lumc.nl)

^3^ Department of Medicine, division of Endocrinology, Leiden University Medical Center, Leiden, The Netherlands

^4^ Leiden Genome Technology Center (LGTC), Department of Human Genetics, Leiden University Medical Center (LUMC), Leiden, The Netherlands

* Correspondence: E-mail: [K.Willems_van_Dijk@lumc.nl](mailto:K.Willems_van_Dijk@lumc.nl);

Table S1. Primer sequences used for PacBio sequencing.

| **Primer name** | **Primer sequence** |
| --- | --- |
| M13-F-GM3F | TGTAAAACGACGGCCAGTAGAGTTTGATCMTGGC |
| M13-F-GM3F-YM | TGTAAAACGACGGCCAGTAGAGTTTGATYMTGGC |
| M13-R-GM4R | CAGGAAACAGCTATGACCTACCTTGTTACGACTT |
| Universal Tail Forward | CCATC\|16-nt-barcode\|TGTAAAACGACGGCCAGT |
| Universal Tail Reverse | GGTAG\|16-nt-barcode\|CAGGAAACAGCTATGACC |

Table S2. Statistics of data sets

| **Group** | **Data set** | **Sample** | **Read Count** | **Joined reads** | **Mean read length (bp)** | **Total number of bases (bp)** |
| --- | --- | --- | --- | --- | --- | --- |
| Control | FL | C1 | 19389 | - | 1514 | 29354586 |
|  | FL | C2 | 9404 | - | 1494 | 14052243 |
|  | V4 PacBio | C1 | 9288 | - | 253 | 2347424 |
|  | V4 PacBio | C2 | 4061 | - | 251 | 1019710 |
|  | V4 Illumina | C1 | 240682 | 109650 | 253 | 27738445 |
|  | V4 Illumina | C2 | 304190 | 142428 | 253 | 36025327 |
| Inulin | FL | In1 | 20986 | - | 1518 | 31863815 |
|  | FL | In2 | 7782 | - | 1505 | 11714148 |
|  | V4 PacBio | In1 | 9799 | - | 253 | 2475902 |
|  | V4 PacBio | In2 | 3386 | - | 251 | 850296 |
|  | V4 Illumina | In1 | 330802 | 151169 | 253 | 38200080 |
|  | V4 Illumina | In2 | 430798 | 202756 | 253 | 51215941 |
